# Supplementary material for: New composite phenotypes enhance chronic kidney disease classification and genetic associations
Source: PLoS Genet. 2025 May 23;21(5):e1011718. doi: 10.1371/journal.pgen.1011718 (PMC12133187; doi:10.1371/journal.pgen.1011718)
Supplement: S1 Table — (DOCX) [file pgen.1011718.s001.docx]

**S1 Table. Trait loadings for the CP extracted from {eGFR, CYSC, ALB, HbA1c, GGT, LDL, and MA}.**

| Phenotype | PC1 (The composite phenotype) | PC2 |
| --- | --- | --- |
| ALB | 0.227 | 0.521 |
| CYSC | -0.649 | 0.027 |
| eGFR | 0.598 | 0.102 |
| GGT | -0.149 | 0.613 |
| HbA1c | -0.319 | 0.185 |
| LDL | 0.103 | 0.457 |
| MA | -0.187 | 0.313 |
