# Supplementary material for: New composite phenotypes enhance chronic kidney disease classification and genetic associations
Source: PLoS Genet. 2025 May 23;21(5):e1011718. doi: 10.1371/journal.pgen.1011718 (PMC12133187; doi:10.1371/journal.pgen.1011718)
Supplement: S1 Text — (DOCX) [file pgen.1011718.s002.docx]

Urine albumin measurements were recorded for all 484,832 participants. However, only 159,364 participants had detectable urine albumin levels, while the remaining individuals had values below the detection limit. In our analysis, to address the issue of urine albumin levels below the detection threshold of 6.7 mg/L for a significant number of participants, we implemented a multi-step procedure. First, urine albumin levels flagged as “<6.7” were set to 6.7 mg/L. Next, the urine albumin-to-creatinine ratio (UACR) was calculated by dividing urine albumin by urine creatinine, and microalbuminuria (MA), which was used as one of the CKD-related biomarkers in our study, was defined as UACR > 30 mg/g.

To mitigate potential bias introduced by with the urine albumin levels, we excluded participants with UACR > 30 mg/g whose urine albumin levels were below the detection limit (6.7 mg/L). This exclusion was crucial to avoid overestimating MA prevalence caused by low urine creatinine levels inflating UACR despite undetectable albumin levels. Although this step may exclude valid cases of MA in individuals with genuinely low creatinine, it ensures the robustness of MA classification and minimizes the risk of bias in the analysis.

The table below provides an additional explanation for why it was necessary to remove samples with urine albumin < 6.7 mg/L and UACR > 30 mg/g.

Table 1. Classification of participants based on Urine Albumin, Urine Creatinine, and UACR.

| Scenario | Urine Albumin | Urine Creatinine | UACR | Reason for Inclusion/Exclusion |
| --- | --- | --- | --- | --- |
| 1. Normal, no MA | >6.7 mg/L | Normal | <30 mg/g | Included: Normal physiological range. |
| 2. MA with detectable albumin | >6.7 mg/L | Low | >30 mg/g | Included: Clear case of MA. |
| 3. Below detection albumin, no MA | <6.7 mg/L | Normal or high | <30 mg/g | Included: Non-MA participant, consistent with healthy population norms. |
| 4. Below detection albumin, borderline MA | <6.7 mg/L | Very low | >30 mg/g | Excluded: Likely an artifact caused by low creatinine inflating UACR. |
| 5. Below detection albumin, true MA | <6.7 mg/L | Extremely low | >30 mg/g | Excluded: Risk of removing a true MA case, but necessary to avoid bias. |
