# Supplementary material for: New composite phenotypes enhance chronic kidney disease classification and genetic associations
Source: PLoS Genet. 2025 May 23;21(5):e1011718. doi: 10.1371/journal.pgen.1011718 (PMC12133187; doi:10.1371/journal.pgen.1011718)
Supplement: S2 Text — (DOCX) [file pgen.1011718.s003.docx]

**S1. R script used in cPCA.**

| # Load required libraries  library(FactoMineR) # For PCA analysis  library(caret) # For machine learning utilities  library(pROC) # For calculating ROC and AUC  library(tidyr) # For data manipulation  library(corrplot) # For correlation plots  library(ggplot2) # For data visualization  # Load cleaned phenotype data without missing values  pheno <- read.table("CKDpheno-imputedtraits.txt", header = TRUE)  head(pheno) # Display the first few rows of phenotype data  # Load CKD-related clinical outcome data  outcome <- read.table("health_related_outcomes_ckd_types.txt", header = TRUE)  sort(colSums(outcome)) # Sort outcomes by their total sums  # Generate a binary CKD indicator (1 for presence of any CKD, 0 otherwise)  ckd <- ifelse(rowSums(outcome) > 0, 1, 0)  outcome$ckd <- ckd # Add CKD indicator to outcome data  # Merge clinical outcomes and phenotype data  dat <- merge(outcome[ncol(outcome)], pheno, by = 0) # Merge on row names  rownames(dat) <- dat$Row.names # Set row names from the merge result  dat <- dat[-1] # Remove unnecessary column  head(dat) # Display the first few rows of merged data  # Split data into training and test sets (70% training, 30% testing)  set.seed(55) # Set seed for reproducibility  sample <- sample(c(TRUE, FALSE), nrow(dat), replace = TRUE, prob = c(0.7, 0.3))  train <- dat[sample, ] # Training set  test <- dat[!sample, ] # Test set  # Generate all combinations of phenotypes, from 2 to 21 phenotypes per combination  phenotypes <- names(dat)[2:ncol(dat)]  combinations <- c()  for (i in 2:21) {  combinations <- c(combinations, combn(phenotypes, i, simplify = FALSE))  }  length(combinations) # Total number of combinations  head(combinations, 30) # Display the first 30 combinations  # Note: accessing a specific combination (example index 2097130)  combinations[2097130]  # Initialize a data frame to store results of each combination's AUC  tab <- as.data.frame(matrix(ncol = 2))  names(tab) <- c("comb", "auc")  # Loop through a range of combinations, calculate AUC for each in the training set  for (i in mem:1100000) { # Adjust 'mem' as the starting point for continuation  print(i) # Display current index in loop  # Get the current combination of phenotypes  comb <- unlist(combinations[i])  comb.name <- paste(comb, collapse = "_") # Create a name for the combination  # Subset training data for the selected combination of phenotypes  sdat <- train[comb]  # Perform PCA on the subset data  pca <- PCA(sdat, ncp = 1, graph = FALSE)  pc <- as.data.frame(pca$ind$coord) # Get the principal component scores  # Calculate the AUC for the first principal component  auc <- roc(train[, 1], pc[, 1])$auc  tab <- rbind(tab, c(comb.name, round(auc, 3))) # Store combination and AUC  }  # Save the current index for future continuation if needed  mem <- i  # Write the results to an output file, with the current index included in the filename  write.table(tab, paste0("cPCA.out.", mem, ".txt"), row.names = FALSE, quote = FALSE) |
| --- |

**S2. Bash scripts used in the study.**

**S2.1. Phenotype prepration**

| #!/bin/bash  ### Prepare the phenotype file for plink2 gwas: have the #FID and IID columns followed by phenotype columns  ### The input file must be a space-delimited file with its first column to be eid and the phenotypes with names in the following columns  file=$1  name=`awk -F'.' '{print $1}' <<< $file`  awk '{print $1,$0}' $file \| sed 's/eid eid/#FID IID/g' > $name.plink2 |
| --- |

| #!/bin/bash  ### Run glm function from plink2  plink2=/home/n10611479/src/plink2_linux_avx2_20220121/plink2  file=$1  pheno=$2  for i in {1..22} ; do  $plink2 --pfile ~/ukbiobank/geno/qced/ukb22828_c${i}_b0_v3_qced \  --glm cols=chrom,pos,ref,alt,a1freq,firth,test,nobs,orbeta,se,ci,tz,p hide-covar \  --pheno $file \  --pheno-name $pheno \  --covar ~/ukbiobank/geno/meta/UKBB_age_sex_20GeneticPCs.plink2 \  --memory 30000 \  --threads 16 \  --no-input-missing-phenotype \  --out plink2.out/chr$i  done |
| --- |

**S2.2. GWAS**

**S2.3. LDSC summary statistics munging**

| #!/bin/bash  # Convert the summary statistics for ldsc analyses  source activate ldsc  ldsc_path=/home/n10611479/src/ldsc  for trait in `echo $1 \| tr ',' ' '` ; do  $ldsc_path/munge_sumstats.py \  --sumstats /home/n10611479/ukbiobank/220119Redo/plink2.out/chrall/chrall.${trait}.glm \  --out ${trait}-munged \  --merge-alleles $ldsc_path/w_hm3.snplist \  --snp ID \  --N-col OBS_CT \  --a1 REF \  --a2 ALT \  --frq A1_FREQ \  --ignore CHROM,POS,SE,T_STAT,A1 \  --chunksize 500000  done |
| --- |

**S2.4. Calculate SNP-based heritability**

| #!/bin/bash  # Calculate snp-based h2  source activate ldsc  ldsc_path=/home/n10611479/src/ldsc  for trait in `echo $1 \| tr ',' ' '` ; do  $ldsc_path/ldsc.py \  --h2 ${trait}-munged.sumstats.gz \  --ref-ld-chr $ldsc_path/baselineLD_v1.1/baselineLD. \  --w-ld-chr $ldsc_path/1000G_Phase3_weights_hm3_no_MHC/weights.hm3_noMHC. \  --frqfile-chr $ldsc_path/1000G_Phase3_frq/1000G.EUR.QC. \  --out h2/${trait}-h2  done |
| --- |

**S2.5. Calculate genetic correlation between a pair of phenotypes**

| #!/bin/bash  # Calculate genetic correlation between pairs of traits  source activate ldsc  ldsc_path=/home/n10611479/src/ldsc  traits=`echo $1 \| tr ',' ' '`  for trait1 in $traits ; do  for trait2 in $traits ; do  if [ "$trait1" == "$trait2" ] \|\| [[ -f rg/$trait1.$trait2.rg.log ]] \|\| [[ -f rg/$trait2.$trait1.rg.log ]] ; then  continue  else  $ldsc_path/ldsc.py \  --rg $trait1-munged.sumstats.gz,$trait2-munged.sumstats.gz \  --ref-ld-chr $ldsc_path/baselineLD_v1.1/baselineLD. \  --w-ld-chr $ldsc_path/1000G_Phase3_weights_hm3_no_MHC/weights.hm3_noMHC. \  --out rg/$trait1.$trait2.rg  fi  done  done |
| --- |
