# Supplementary material for: New composite phenotypes enhance chronic kidney disease classification and genetic associations
Source: PLoS Genet. 2025 May 23;21(5):e1011718. doi: 10.1371/journal.pgen.1011718 (PMC12133187; doi:10.1371/journal.pgen.1011718)
Supplement: Attachment — Submitted filename: Response_to_reviewers.docx [file pgen.1011718.s005.docx]

**Response to reviewers**

**Reviewer #1:** I commend the authors on this undertaking and for writing a clear and concise article looking at combinations of biomarkers and genetic hits in CKD. It was a pleasure to read.
While the findings themselves may not be that unexpected, the approach of using PCA to look at biomarkers in a combinatorial manner is a 'why didn't I think of that' moment. It's straightforward, clear, and addresses a lot of issues that arise from using more complex machine learning approaches and should have broad applicability across other large cohort studies - and perhaps in smaller, deeply phenotyped ones as well.

Thank you for your thoughtful and encouraging feedback. We are especially glad to hear that you see potential for broad applicability in both large cohort studies and smaller, deeply phenotyped datasets. We hope this method can serve as a valuable tool for uncovering novel insights in other complex diseases as well. Thank you again for your kind words and for taking the time to review our work.

**Reviewer #2:**

The study by Tran et al. aims to address the limitations of using single biomarkers to assess Chronic Kidney Disease (CKD) by applying combinatorial Principal Component Analysis (cPCA). Using UK Biobank data, the investigators analyzed 21 CKD-related phenotypes, generating over 2 million composite phenotypes (CPs). The top-ranked CP achieving an AUC of 0.878, outperforming eGFR alone (AUC: 0.83). Genetic association analysis identified all major eGFR-associated loci and the SH2B3 locus rs318450, a loss-of-function variant. The study highlights the effectiveness of the cPCA approach in understanding CKD's genetic basis and suggests potential applications for other complex diseases.

This is a novel and interesting approach to dealing with multiple phenotypes. The application to the example of chronic kidney disease is appropriate, although the “gold-standard” definition used here is not conventional nor is it fully adequate. However, the method can be useful in settings such as these.

- The abstract states that the SH2B3 locus has a combined beta of -0.046, with no units associated. Please allow the reader to understand how to interpret that effect estimate without having to read the whole manuscript.

Thank you for your feedback. We agree that providing clearer context for the effect estimate in the abstract would improve readability. Since eGFR was inverse-normal transformed before GWAS, the beta coefficient for the *SH2B3* locus (rs3184504) is unitless. However, to enhance interpretability, we analyzed the relationship between composite phenotypes (CPs) and CKD. The CP analyzed in this analysis was derived from the top-ranked biomarker combination (albumin, cystatin C, eGFR, gamma-glutamyltransferase, HbA1c, low-density lipoprotein, and microalbuminuria), which exhibited the highest classification performance.

Using a logistic regression model predicting CKD status: CKD status ~ CP + age + sex

In this model, the odds ratio (OR) for CP was 0.419 (95% CI:. 0.413 – 0.425). This indicates that each one-unit increase in CP is associated with a 58.1% decrease in CKD odds. For *SH2B3* rs3184504, the effect allele T had a β of -0.046 in the GWAS of PC1, meaning that each additional copy of the T allele is associated with a decrease in CP. Since higher CP values are associated with lower CKD odds, a decrease in CP due to *SH2B3* suggests an increase risk of CKD. This finding aligns with previous studies showing the T allele is associated with increased blood pressure, immune activation, and kidney dysfunction [1].

We have revised the abstract to: “Genetic association analysis of the ~50,000 high-performing CPs identified all major eGFR-associated loci, except for the *SH2B3* locus rs3184504-T, a loss-of-function variant with a damaging effect on CKD, which was uniquely identified in CPs (p = 3.1 × 10⁻⁵⁶) but not in eGFR within the same sample size” (See abstract page 2, lines 30-33).

In addition, we have also added the relationship between the CP and CKD and a PCA biplot to the Results section (pages 9-10, lines 157-166). The PCA biplot visually represents the contributions of each biomarker and the separation between CKD cases and non-cases along the composite phenotype.

**Figure 1.** PCA biplot illustrating the PCA scores and the trait loadings on the leading cPC1 extracted from eGFR, CYSC, MA, HbA1c, LDL, ALB, and GGT. Samples were labeled with chronic kidney disease status (1:case; 0:non-case).

- Could the investigators also show how a plot similar to Figure 2 but with CKD instead of

eGFR.

Thank you for this suggestion. We have generated a new figure illustrating the 80 loci identified across ~50,000 CPs and their overlap with loci identified in CKD. In this analysis, 18 loci reached genome-wide significance (p < 5×10⁻⁸) for CKD.

**Figure 2.** Venn diagram showing the 80 genetic loci identified across ~50,000 CPs and their overlap with loci identified in CKD.

- It would be helpful to show how this approach improves upon other PCA methods, if at all.

Thank you for your question. The key purposes of cPCA are:

1. Identifying optimal combinations of biomarkers that collectively offer improved discriminatory power in disease classification, outperforming individual biomarkers alone.

2. Identifying genetic loci associated with the target disease, leveraging multi-trait GWAS approaches.

For the second objective, cPCA shares similarities with multivariate GWAS and other joint analyses of multiple traits, including PCA-based methods (e.g., combined-PC [2] or adaptive principle component test [3]). However, unlike traditional multivariate methods that require a predefined set of traits, cPCA systematically explores and selects optimal biomarker combinations, making it a distinct and more flexible approach.

We have now added this response to the Discussion section (page 15, lines 245–252) to highlight the novelty of the cPCA approach.

Also, what are the loadings of the different biomarkers in each PC?

Thank you for your question. We have included a table of trait loadings for the top-ranked CP (Table 1). The individual biomarkers contributed differently to the leading CP, cystatin C and eGFR showing the highest loadings, followed by HbA1c, albumin, and microalbuminuria. Gamma-glutamyltransferase and low-density lipoprotein had comparatively lower loadings. This table has now been added to the manuscript as S1 Table.

**Table 1.** Trait loadings for the CP extracted from {eGFR, CYSC, ALB, HbA1c, GGT, LDL, and MA}.

| Phenotype | PC1 (The composite phenotype) | PC2 |
| --- | --- | --- |
| ALB | 0.227 | 0.521 |
| CYSC | -0.649 | 0.027 |
| eGFR | 0.598 | 0.102 |
| GGT | -0.149 | 0.613 |
| HbA1c | -0.319 | 0.185 |
| LDL | 0.103 | 0.457 |
| MA | -0.187 | 0.313 |

- The “gold standard” against which the biomarker combinations are being tested is CKD defined by ICD-code. The ICD-codes chosen for this definition have notoriously low sensitivity (around 25-30%) and varying specificity. Because ICD codes for CKD underestimate the true incidence and prevalence of CKD, with moderate cases of CKD particularly underreported, this study may be inadvertently selecting individuals with more severe disease, which may limit the generalizability of the findings and should be acknowledged as a limitation. This may also explain why some of the loci found to be associated with CKD but not in the original eGFR GWAS are linked to comorbidities (e.g. FTO – obesity, SH2B3 - diabetes).

Thank you for your insightful comment. We acknowledge that ICD codes for CKD have limitations in sensitivity, leading to potential underreporting of CKD cases. This is an important consideration, and we agree that it may introduce selection bias toward individuals with more severe disease, which could limit the generalizability of our findings. We have now explicitly acknowledged this as a study limitation in the manuscript (page 17, lines 313-323). Nevertheless, the use of ICD-based CKD diagnoses remains common in large-scale biobank studies, particularly when serum creatinine-based eGFR values are unavailable for long-term CKD classification. While this approach likely enriches the cohort for more advanced CKD cases, it maintains high specificity—reported between 82% and 99%—ensuring that cases included in our analysis truly represent CKD rather than transient reductions in kidney function [4, 5].

- Microalbuminuria is only available in 156K participants in the UK Biobank. Were the remaining 200K imputed? Please elaborate on the imputation details.

Thank you for your question. We would like to clarify that urine albumin measurements were recorded for all 484,832 participants. However, only 159,364 participants had detectable urine albumin levels, while the remaining individuals had values below the detection limit. This is expected, as urine albumin levels are typically undetectable in a healthy population.

In our analysis, to address the issue of urine albumin levels below the detection threshold of 6.7 mg/L for a significant number of participants, we implemented a multi-step procedure. First, urine albumin levels flagged as “<6.7” were set to 6.7 mg/L. Next, the urine albumin-to-creatinine ratio (UACR) was calculated by dividing urine albumin by urine creatinine, and microalbuminuria (MA), which was used as one of the CKD-related biomarkers in our study, was defined as UACR > 30 mg/g.

To mitigate potential bias introduced by with the urine albumin levels, we excluded participants with UACR > 30 mg/g whose urine albumin levels were below the detection limit (6.7 mg/L). This exclusion was crucial to avoid overestimating MA prevalence caused by low urine creatinine levels inflating UACR despite undetectable albumin levels. Although this step may exclude valid cases of MA in individuals with genuinely low creatinine, it ensures the robustness of MA classification and minimizes the risk of bias in the analysis.

Please also refer to the table below, which provides an additional explanation for why it was necessary to remove samples with urine albumin < 6.7 mg/L and UACR > 30 mg/g.

**Table 2.** Classification of participants based on Urine Albumin, Urine Creatinine, and UACR.

| Scenario | Urine Albumin | Urine Creatinine | UACR | Reason for Inclusion/Exclusion |
| --- | --- | --- | --- | --- |
| 1. Normal, no MA | >6.7 mg/L | Normal | <30 mg/g | Included: Normal physiological range. |
| 2. MA with detectable albumin | >6.7 mg/L | Low | >30 mg/g | Included: Clear case of MA. |
| 3. Below detection albumin, no MA | <6.7 mg/L | Normal or high | <30 mg/g | Included: Non-MA participant, consistent with healthy population norms. |
| 4. Below detection albumin, borderline MA | <6.7 mg/L | Very low | >30 mg/g | Excluded: Likely an artifact caused by low creatinine inflating UACR. |
| 5. Below detection albumin, true MA | <6.7 mg/L | Extremely low | >30 mg/g | Excluded: Risk of removing a true MA case, but necessary to avoid bias. |

We have now added this response to the supporting text S1.

- Typo on page 5, line 90 – “non-significant”. Beyond the typo issue – it would be more helpful to give the actual p-value rather than p>0.05.

Thank you for your feedback. We have corrected the typo. In addition, instead of stating p > 0.05, we now provide the precise p-value. The revised text now reads:*“*Although the AUC value of CYSC was slighly higher than that of eGFR, comparing the 2 ROC curves showed no statistically significant difference (p=0.256)” (page 5, line 101).

- Typo on page 12, line 178 – should be “eGFR” instead of CYSC, no?

Thank you for catching this error. We have changed from “CYSC” to “eGFR” in the revised version (page 14, line 243).

**Reviewer #3:** Tran et al. proposed a combinatorial Principal Component Analysis (cPCA) to analyze the combination of different etiologies that lead to loss of kidney function. The top-ranked principal components showed higher classification power for clinical chronic kidney disease. However, it is not clear what are the biological insights delivered in this study. Some major issues are listed in the following:

1. The phenotypes used are not independent of each other, which may introduce bias in the quantification of the combinational effects of chronic kidney disease.

Thank you for pointing out the concern regarding potential bias due to non-independence among the phenotypes used. We acknowledge that some of the phenotypes, such as BUN and UA, are highly correlated with eGFR, which could introduce and bias when analyzing combinational effects. However, the combinatorial Principal Component Analysis (cPCA) inherently accounts for multicollinearity and prioritizes phenotypes that contribute unique and independent information.

Specifically, CYSC, eGFR, HbA1c, MA, ALB, LDL, and GGT were the most frequently observed phenotypes, appearing in 75% to 48% of the ~50,000 identified combinations. Importantly, phenotypes like BUN and UA, which are strongly correlated with eGFR and demonstrated higher performance in CKD classification, were less frequently selected (BUN: 22.4%, UA: 0%). This observation suggests that cPCA effectively mitigated redundancy by favoring phenotypes that are less collinear and more independent in their contributions to the analysis.

This response was also a part of the Discussion (page 15, lines 257-262).

2. Does the study identify a new variant associated with kidney function or chronic kidney disease? For example, the example variant (rs3184504, there is a typo rs318450 in the abstract) has been identified as a variant associated with eGFR in previous GWAS studies.

Thank you for pointing out the typographical error in the abstract, which we have now corrected in the revised manuscript, and for your question regarding the identification of novel variants. The variant highlighted in our study, rs3184504, has indeed been previously reported in large, well-powered GWAS with sample sizes of approximately 1 million individuals. In our study this variant was robustly identified using a comparatively smaller cohort (~300,000 subjects) and successfully replicated in an even smaller sample size (n = 11,206). This finding emphasizes the increased power and effectiveness of the method (cPCA) in identifying genetic associations for complex phenotypes like CKD.

This response has now been added to the Discussion (page 16, lines 285-290).

3. The conclusions in this study need to be validated by independent methods. For example, colocalization analysis of multiple traits is a powerful tool for exploring the combinational contribution of multiple traits. It would be useful to compare the preformation of cPCA and colocalization analysis with this aim.

Thank you for your suggestion regarding the validation of our conclusions using independent methods, such as colocalization analysis. As recommended, we applied coloc to assess whether the genetic associations identified using CPs share the same causal variants as those detected in the traditional eGFR GWAS.

Our analysis revealed that among the 2 loci identified uniquely through the multi-phenotype cPCA approach and subsequent replication analysis, i.e. *CST3* and *SH2B3*, *SH2B3* showed strong evidence of colocalization with eGFR, supporting its role in kidney function. In contrast, *CST3* was associated with composite phenotypes but did not share a causal variant with eGFR. As discussed, the *CST3* signal was likely driven by the presence of CYSC in nearly all composite phenotype combinations. Since *CST3* was not genome-wide significant in any other individual-trait GWAS except for CYSC, this suggests that *CST3* functions primarily as a biomarker gene rather than a causal gene influencing CKD pathology.

**Table 3.** Colocalization probabilities for each locus are presented across five categories. Probabilities range from 0 to 1, with higher values indicating stronger support for a given colocalization scenario.

| Loci | No causal variant | Causal variant for the CP only | Causal variant for eGFR only | Two distinct causal variants | One common causal variant |
| --- | --- | --- | --- | --- | --- |
| *SH2B3* | 1.39E-39 | 0.1745 | 7.12E-41 | 0.008 | **0.817** |
| *CST3* | 3.41E-291 | **0.970** | 3.66E-293 | 0.010 | 0.020 |

These results have been added to the Results section (pages 13-14, lines 216-228).

4. What’s the rule of the order (No.) of phonotypes is in Table 1? It would be useful to order the traits based on the AUC.

Thank you for your suggestion regarding the ordering of phenotypes in Table 1. Initially, the phenotypes were listed in alphabetical order for simplicity. However, as you suggested, we have now re-ordered them based on their AUC values. This revised order better highlights the most important biomarkers in CKD classification, making it easier to identify the phenotypes with the highest predictive performance (See Table 1, pages 5-6).

**Reference**

1.	Alexander MR, Hank S, Dale BL, Himmel L, Zhong X, Smart CD, et al. A Single Nucleotide Polymorphism in SH2B3/LNK Promotes Hypertension Development and Renal Damage. Circ Res. 2022;131(9):731-47.

2.	Aschard H, Vilhjálmsson Bjarni J, Greliche N, Morange P-E, Trégouët D-A, Kraft P. Maximizing the Power of Principal-Component Analysis of Correlated Phenotypes in Genome-wide Association Studies. The American Journal of Human Genetics. 2014;94(5):662-76.

3.	Bu D-l, Zhang S-g, Li N. Analyzing Multiple Phenotypes Based on Principal Component Analysis. Acta Mathematicae Applicatae Sinica, English Series. 2022;38(4):843-60.

4.	Paik JM, Patorno E, Zhuo M, Bessette LG, York C, Gautam N, et al. Accuracy of identifying diagnosis of moderate to severe chronic kidney disease in administrative claims data. Pharmacoepidemiol Drug Saf. 2022;31(4):467-75.

5.	Bothe T, Fietz AK, Schaeffner E, Douros A, Pöhlmann A, Mielke N, et al. Diagnostic Validity of Chronic Kidney Disease in Health Claims Data Over Time: Results from a Cohort of Community-Dwelling Older Adults in Germany. Clin Epidemiol. 2024;16:143-54.
