# Supplementary material for: New composite phenotypes enhance chronic kidney disease classification and genetic associations
Source: PLoS Genet. 2025 May 23;21(5):e1011718. doi: 10.1371/journal.pgen.1011718 (PMC12133187; doi:10.1371/journal.pgen.1011718)
Supplement: Attachment — Submitted filename: Response_to_reviewers_auresp_2.docx [file pgen.1011718.s006.docx]

**Response to reviewers**

**Reviewer #3:**

The only remained concern is "2. Does the study identify a new variant associated with kidney function or chronic kidney disease?". If there is new variant, please list them. If not, please also list the previous studies which have identified each variant, as an validation. This information is necessary to illustrate the importance of these significant variants.

Thank you for your question. To validate the identified variants, we compared them against the largest GWAS of creatinine-based eGFR conducted in 1.2 million individuals [1]. Overall, the effect directions were consistent across the five loci. However, only the *SH2B3* locus reached genome-wide significance (p ≤ 5×10⁻⁸). Although not genome-wide significant, the *SEMA3F-AS1* and *AC128707.1* loci reached suggestive significance thresholds (p ≤ 1×10⁻⁵), while the *CST3* and *FTO* loci showed no evidence of association (p ≥ 0.05).

Table. Validation of the five loci identified by the cPCA method in the GWAS meta-analysis of creatinine-based eGFR.

| **Gene** | **rsID** | **Marker Name** | **GWAS meta-analysis for eGFR (n=1,201,909)** | | |
| --- | --- | --- | --- | --- | --- |
|  |  |  | **Beta** | **SE** | **P** |
| *CST3* | rs2405392 | 20:23569186:C:T | 6.00×10^-4^ | 3.00×10^-4^ | 0.04875 |
| *SH2B3* | rs3184504 | 12:111884608:C:T | -0.0016 | 3.00×10^-4^ | 6.72×10^-10^ |
| *FTO* | rs56313538 | 16:53818834:A:G | -2.00×10^-4^ | 3.00×10^-4^ | 0.534 |
| *SEMA3F-AS1* | rs2624847 | 3:50174197:T:G | -0.0014 | 3.00×10^-4^ | 4.86×10^-6^ |
| *AC128707.1* | rs7311712 | 12:78807411:C:T | 0.0013 | 3.00×10^-4^ | 5.70×10^-7^ |

We have now incorporated these results into the Results section (pages 13-14, lines 183-192).

Reference

1.	Stanzick KJ, Li Y, Schlosser P, Gorski M, Wuttke M, Thomas LF, et al. Discovery and prioritization of variants and genes for kidney function in >1.2 million individuals. Nat Commun. 2021;12(1):4350.
